# Supplementary material for: Developmental Dynamics of the Gut Virome in Tibetan Pigs at High Altitude: A Metagenomic Perspective across Age Groups
Source: Viruses. 2024 Apr 14;16(4):606. doi: 10.3390/v16040606 (PMC11054254; doi:10.3390/v16040606)

Supplementary Figure S1. Venn diagram of results from three virus sequence identification tools

IMG, VirFinder, VirSorter2 were used to identified putitive viral contigs.

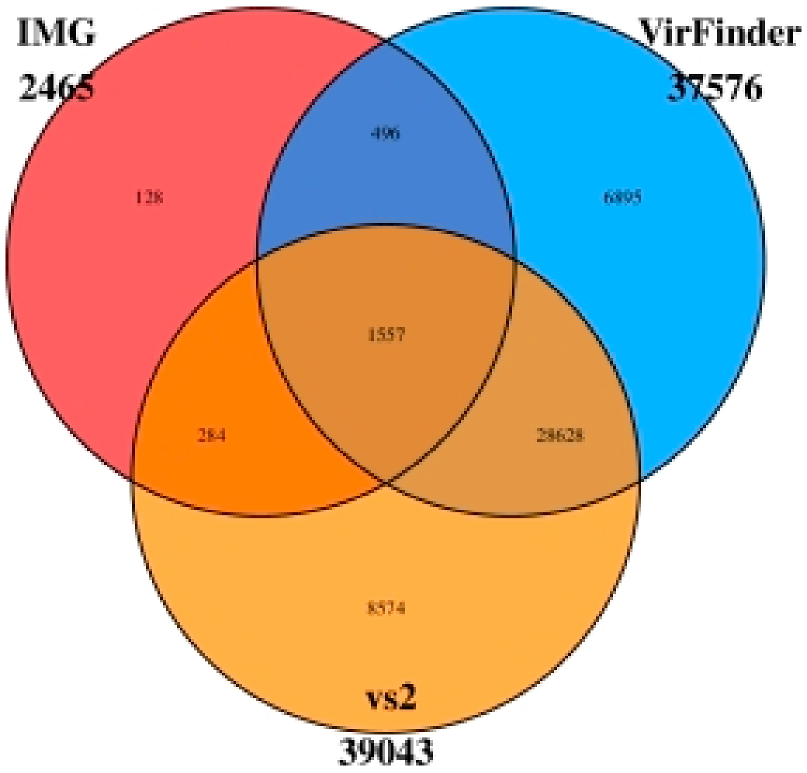

Supplement: Supplementary file 1 [file viruses-16-00606-s001.zip › Supplemental Figure S1.pdf]
